# Supplementary material for: Characterization of Seminal Microbiome of Infertile Idiopathic Patients Using Third-Generation Sequencing Platform
Source: Int J Mol Sci. 2023 Apr 26;24(9):7867. doi: 10.3390/ijms24097867 (PMC10178615; doi:10.3390/ijms24097867)
Supplement: Supplementary file 1 [file ijms-24-07867-s001.zip › Supplementary Figure S1.pdf]

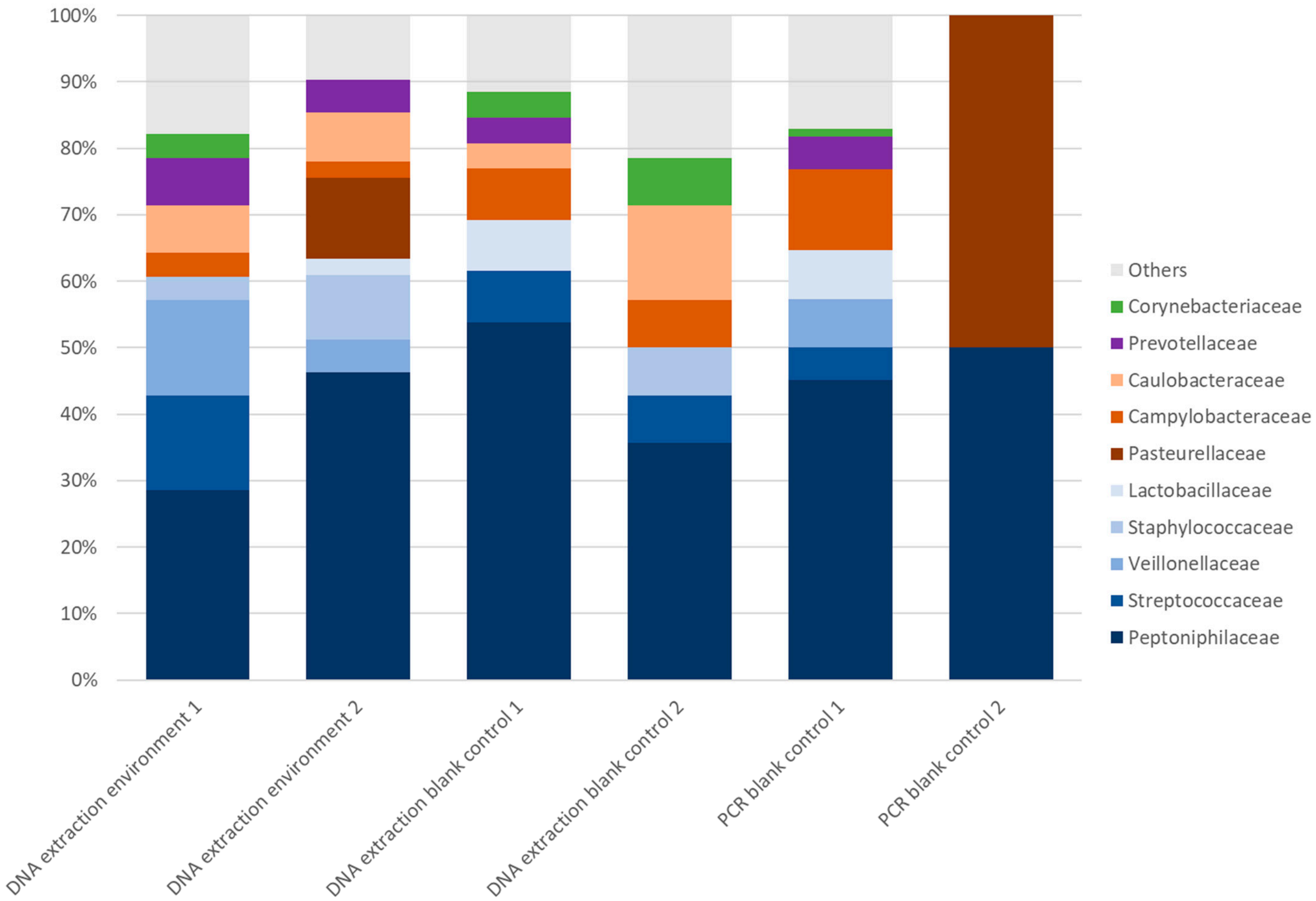

Supplementary Figure S1. Relative abundance in percentage of bacteria detected in negative controls at the family taxonomic level. The X-axis shows each type of negative sample: environmental samples taken from the laminar flux cabin where DNA extraction was processed (DNA extraction environment), blank controls from DNA extraction (DNA extraction blank control) and PCR (PCR blank control) procedures to observe the *kitoma*.
