# Supplementary material for: Characterization of Seminal Microbiome of Infertile Idiopathic Patients Using Third-Generation Sequencing Platform
Source: Int J Mol Sci. 2023 Apr 26;24(9):7867. doi: 10.3390/ijms24097867 (PMC10178615; doi:10.3390/ijms24097867)
Supplement: Supplementary file 1 [file ijms-24-07867-s001.zip › Supplementary Table S1.pdf]

Supplementary Table S1. Relative abundance of taxa identified by 16S rRNA sequencing in semen samples using ONT MinION platform. The mean proportion of each taxon with a minimum relative abundance of 0.05% in whole sample is shown.

	Total (%)	Controls (%)	Idiopathic patients (%)
**Phylum**			
Firmicutes	66.51	71.90	64.72
Proteobacteria	20.81	17.59	21.88
Bacteroidetes	5.37	5.43	5.35
Actinobacteria	5.33	4.93	5.47
Tenericutes	1.54	0.00	2.05
Fusobacteria	0.31	0.13	0.37
Cyanobacteria	0.08	0.00	0.10
**Family**			
Peptoniphilaceae	38.79	44.33	36.94
Staphylococcaceae	7.99	15.62	5.45
Campylobacteraceae	6.34	3.14	7.41
Prevotellaceae	5.65	5.95	5.54
Streptococcaceae	4.88	3.86	5.21
Lactobacillaceae	4.46	0.14	5.90
Veillonellaceae	2.92	2.44	3.08
Bacillaceae	2.46	1.11	2.91
Enterococcaceae	2.42	3.84	1.94
Enterobacteriaceae	2.37	0.08	3.13
Corynebacteriaceae	2.17	2.89	1.93
Burkholderiaceae	1.83	1.04	2.09
Propionibacteriaceae	1.64	1.03	1.85
Mycoplasmataceae	1.64	0.00	2.18
Moraxellaceae	1.48	0.03	1.97
Pasteurellaceae	1.45	5.26	0.17
Actinomycetaceae	1.29	1.01	1.38
Caulobacteraceae	1.12	1.68	0.93
Aerococcaceae	1.04	0.74	1.14
Rhodobacteraceae	0.92	2.06	0.53
Porphyromonadaceae	0.88	0.25	1.10
Oxalobacteraceae	0.81	0.14	1.03
Listeriaceae	0.80	0.17	1.01
Bradyrhizobiaceae	0.52	0.04	0.68
Comamonadaceae	0.43	0.46	0.42
Micrococcaceae	0.43	0.10	0.54
Neisseriaceae	0.42	0.00	0.56
Fusobacteriaceae	0.41	0.14	0.50
Pseudomonadaceae	0.24	0.18	0.26
Sphingomonadaceae	0.24	0.74	0.07
Peptostreptococcaceae	0.23	0.28	0.21
Clostridiaceae	0.21	0.01	0.28
Planococcaceae	0.20	0.01	0.26

Paenibacillaceae	0.16	0.00	0.21
Thermoactinomycetaceae	0.12	0.00	0.15
Bifidobacteriaceae	0.11	0.02	0.14
Tissierellaceae	0.10	0.06	0.12
Methylobacteriaceae	0.09	0.35	0.00
Weeksellaceae	0.08	0.14	0.06
Carnobacteriaceae	0.08	0.05	0.09
Eubacteriales	0.06	0.20	0.02
**Genera**			
Peptoniphilus	15.71	15.49	13.31
Finegoldia	14.74	15.64	12.13
Staphylococcus	8.28	14.32	4.96
Anaerococcus	6.63	7.91	5.16
Campylobacter	6.01	2.89	6.10
Prevotella	5.41	5.36	4.58
Streptococcus	5.34	3.68	5.05
Lactobacillus	4.22	0.03	4.95
Ezakiella	3.78	1.53	3.93
Enterococcus	2.59	3.63	1.83
Corynebacterium	2.28	2.78	1.75
Bacillus	2.20	0.69	2.35
Dialister	1.92	1.58	1.73
Ureaplasma	1.80	0.00	2.11
Ralstonia	1.76	0.91	1.77
Cutibacterium	1.37	0.75	1.36
Moraxella	1.35	0.00	1.59
Haemophilus	1.27	4.15	0.12
Brevundimonas	0.97	1.62	0.60
Massilia	0.93	0.09	1.06
Paracoccus	0.89	1.79	0.45
Porphyromonas	0.85	0.22	0.93
Veillonella	0.82	0.60	0.76
Bradyrhizobium	0.71	0.08	0.80
Listeria	0.65	0.14	0.72
Winkia	0.49	0.24	0.50
Gardnerella	0.43	0.00	0.50
Neisseria	0.42	0.00	0.49
Fusobacterium	0.40	0.13	0.43
Rothia	0.33	0.01	0.38
Mobiluncus	0.29	0.21	0.28
Fenollaria	0.25	0.21	0.23
Varibaculum	0.25	0.25	0.21
Gemella	0.25	0.00	0.29
Pseudomonas	0.25	0.18	0.23
Pelomonas	0.23	0.44	0.13
Clostridium	0.22	0.00	0.26
Facklamia	0.21	0.52	0.07

Peptostreptococcus	0.18	0.19	0.15
Methylobacterium	0.18	0.63	0.00
Delftia	0.16	0.00	0.19
Salmonella	0.16	0.07	0.17
Limosilactobacillus	0.15	0.00	0.17
Rummeliibacillus	0.14	0.00	0.17
Brochothrix	0.14	0.00	0.16
Parvimonas	0.13	0.00	0.15
Planifilum	0.12	0.00	0.14
Paenibacillus	0.12	0.00	0.14
Acinetobacter	0.11	0.03	0.12
Methylorubrum	0.09	0.32	0.00
Aerococcus	0.09	0.02	0.10
Sphingobium	0.08	0.28	0.00
Companilactobacillus	0.08	0.00	0.09
Caulobacter	0.08	0.00	0.09
Chryseobacterium	0.07	0.08	0.06
Kocuria	0.07	0.07	0.05
Macrococcus	0.05	0.04	0.05
