# Supplementary material for: Characterization of Seminal Microbiome of Infertile Idiopathic Patients Using Third-Generation Sequencing Platform
Source: Int J Mol Sci. 2023 Apr 26;24(9):7867. doi: 10.3390/ijms24097867 (PMC10178615; doi:10.3390/ijms24097867)
Supplement: Supplementary file 1 [file ijms-24-07867-s001.zip › Supplementary Table S2.pdf]

Supplementary Table S2. Clustering analysis results for (A) phylum, (B) family and (C) genus taxonomic levels. The mean relative abundance of each taxon in each cluster is shown.

**A** ***PHYLUM***	**Cluster 1**<br>Relative abundance mean	**Cluster 2**<br>Relative abundance mean
Firmicutes	78.51	57.51
Proteobacteria	12.91	26.74
Actinobacteria	6.31	4.60
Bacteroidetes	2.08	7.84
Tenericutes	0.00	2.69

**B** ***FAMILY***	**Cluster 1**<br>Relative abundance mean	**Cluster 2**<br>Relative abundance mean
Peptoniphilaceae	66.92	17.69
Staphylococcaceae	4.63	10.52
Campylobacteraceae	4.13	7.99
Corynebacteriaceae	2.86	1.65
Veillonellaceae	2.75	3.04
Propionibacteriaceae	2.34	1.12
Enterococcaceae	2.27	2.53
Streptococcaceae	1.88	7.12
Prevotellaceae	1.71	8.60
Actinomycetaceae	1.27	1.31
Moraxellaceae	0.77	2.02
Enterobacteriaceae	0.76	3.58
Burkholderiaceae	0.75	2.64
Caulobacteraceae	0.72	1.42
Aerococcaceae	0.64	1.34
Lactobacillaceae	0.17	7.68
Bacillaceae	0.10	4.23
Pasteurellaceae	0.00	2.53
Mycoplasmataceae	0.00	2.86

**C** ***GENUS***	**Cluster 1**<br>Relative abundance mean	**Cluster 2**<br>Relative abundance mean
Peptoniphilus	24.53	5.85
Finegoldia	21.90	6.33
Anaerococcus	10.13	2.63
Staphylococcus	4.31	9.55
Campylobacter	3.67	6.52
Corynebacterium	2.72	1.47
Ezakiella	2.67	3.83
Enterococcus	2.22	2.33
Streptococcus	1.87	6.83
Cutibacterium	1.80	0.76
Prevotella	1.56	7.18
Dialister	0.99	2.22
Ralstonia	0.68	2.21
Moraxella	0.50	1.71
Lactobacillus	0.15	6.41
Bacillus	0.04	3.36
Haemophilus	0.00	1.96
Ureaplasma	0.00	2.77
