# Supplementary material for: Characterization of Seminal Microbiome of Infertile Idiopathic Patients Using Third-Generation Sequencing Platform
Source: Int J Mol Sci. 2023 Apr 26;24(9):7867. doi: 10.3390/ijms24097867 (PMC10178615; doi:10.3390/ijms24097867)
Supplement: Supplementary file 1 [file ijms-24-07867-s001.zip › Supplementary Table S3.pdf]

Supplementary Table S3. Wilcoxon signed-rank test statistic (Z) and p-value of a comparison between the relative abundances obtained by the Illumina platform and those obtained by the ONT platform. A significant result allows discarding the hypothesis that the relative abundance of a given taxon is similar on both platforms. Only taxa above 0.05% global relative abundance on at least one platform were considered.

**PHYLUM**		
	**Z**	**p-valor**
Bacteroidetes	-,571b	0,568
Proteobacteria	-1,379b	0,168
Firmicutes	-2,439c	0,015
Actinobacteria	-3,189b	0,001
Candidatus.Saccharibacteria	-3,516b	0
Cyanobacteria	-4,780b	0
Deinococcus.Thermus	-4,881b	0
delta/epsilon subdivisions	-6,393c	0
Fusobacteria	-3,867b	0
Spirochaetes	-5,424b	0
Tenericutes	-4,484b	0
**FAMILY**		
	**Z**	**p-valor**
Aerococcaceae	-,073c	0,942
Propionibacteriaceae	-,075b	0,94
Campylobacteraceae	-,122b	0,903
Comamonadaceae	-,131c	0,896
Synergistaceae	-,241b	0,809
Caulobacteraceae	-,243b	0,808
Listeriaceae	-,243b	0,808
Porphyromonadaceae	-,338b	0,735
Bacillaceae	-,351c	0,726
Actinomycetaceae	-,452b	0,651
Rhodobacteraceae	-,462b	0,644
Burkholderiaceae	-,486b	0,627
Veillonellaceae	-,486c	0,627
Rhizobiaceae	-,516b	0,606
Prevotellaceae	-,620c	0,535
Oscillospiraceae	-,654b	0,513
Streptococcaceae	-,742b	0,458
Xanthomonadaceae	-,746b	0,456
Planococcaceae	-1,202b	0,229
Chitinophagaceae	-1,224b	0,221
Tissierellaceae	-1,289b	0,197
Pasteurellaceae	-1,557b	0,12
Treponemataceae	-1,604c	0,109
Peptoniphilaceae	-1,631c	0,103
Phyllobacteriaceae	-1,814b	0,07
Corynebacteriaceae	-1,819b	0,069
Sutterellaceae	-1,816b	0,069
Methylobacteriaceae	-1,823b	0,068
Hymenobacteraceae	-1,891b	0,059

Rhodocyclaceae	-1,955c	0,051
Lactobacillaceae	-2,007b	0,045
Steroidobacteraceae	-2,018b	0,044
Bradyrhizobiaceae	-2,041b	0,041
Enterobacteriaceae	-2,162b	0,031
Micrococcaceae	-2,296b	0,022
Peptococcaceae	-2,346b	0,019
Psychromonadaceae	-2,366c	0,018
Oxalobacteraceae	-2,439b	0,015
Yersiniaceae	-2,490b	0,013
Azospirillaceae	-2,521c	0,012
Staphylococcaceae	-2,504c	0,012
Sphingomonadaceae	-2,551b	0,011
Carnobacteriaceae	-2,677c	0,007
Pseudomonadaceae	-2,804b	0,005
Moraxellaceae	-2,874b	0,004
Enterococcaceae	-2,949c	0,003
Intrasporangiaceae	-3,145b	0,002
Fusobacteriaceae	-3,316b	0,001
Roseobacteraceae	-3,296c	0,001
Sinobacteraceae	-3,195b	0,001
Acidiferrobacteraceae	-4,106b	0
Alcaligenaceae	-3,911b	0
Alteromonadaceae	-5,156b	0
Atopobiaceae	-5,485b	0
Bacteroidaceae	-5,358b	0
Bifidobacteriaceae	-4,591b	0
Clostridiaceae	-5,076b	0
Deinococcaceae	-4,783b	0
Dermabacteraceae	-3,828b	0
Erwiniaceae	-4,570b	0
Erysipelotrichaceae	-5,777b	0
Eubacteriaceae	-3,710b	0
Flavobacteriaceae	-5,968b	0
Gottschalkiaceae	-4,785b	0
Hungateiclostridiaceae	-5,012b	0
Lachnospiraceae	-5,209b	0
Leptotrichiaceae	-3,746b	0
Microbacteriaceae	-4,808b	0
Mycoplasmataceae	-4,040b	0
Neisseriaceae	-4,041b	0
Nocardiaceae	-3,745b	0
Paenibacillaceae	-4,662b	0
Peptostreptococcaceae	-4,645b	0
Piscirickettsiaceae	-4,984b	0
Promicromonosporaceae	-4,228b	0
Selenomonadaceae	-4,253b	0
Spirochaetaceae	-5,777b	0
Thermoactinomycetaceae	-3,718b	0
Thermoanaerobacteraceae	-6,215b	0

Weeksellaceae	-3,621c	0
**GENUS**		
	**Z**	**p-valor**
Prevotella	-,125c	0,901
Haemophilus	-,262b	0,793
Porphyromonas	-,308b	0,758
Delftia	-,338c	0,735
Rummeliibacillus	-,341c	0,733
Listeria	-,428c	0,669
Ezakiella	-,436b	0,663
Kocuria	-,449b	0,654
Dialister	-,603b	0,546
Bacillus	-,620b	0,535
Streptococcus	-,783b	0,434
Campylobacter	-,856b	0,392
Anaerococcus	-,873b	0,383
Finegoldia	-,914b	0,361
Brochothrix	-,966b	0,334
Rothia	-1,156b	0,248
Peptoniphilus	-1,191c	0,234
Veillonella	-1,385b	0,166
Massilia	-1,430b	0,153
Sphingomonas	-1,431b	0,153
Brevundimonas	-1,471c	0,141
Chryseobacterium	-1,486b	0,137
Cupriavidus	-1,564b	0,118
Ralstonia	-1,568c	0,117
Bradyrhizobium	-1,608c	0,108
Cutibacterium	-1,625b	0,104
Paracoccus	-1,675c	0,094
Caulobacter	-1,765c	0,078
Methylorubrum	-1,836c	0,066
Janibacter	-1,886b	0,059
Methylobacterium	-1,898b	0,058
Moraxella	-1,925b	0,054
Corynebacterium	-2,023b	0,043
Staphylococcus	-2,113c	0,035
Ureaplasma	-2,136b	0,033
Aerococcus	-2,195b	0,028
Mobiluncus	-2,338b	0,019
Enterococcus	-2,371c	0,018
Planifilum	-2,366c	0,018
Pseudomonas	-2,989b	0,003
Actinotignum	-3,056b	0,002
Filifactor	-3,459b	0,001
Flavobacterium	-3,406b	0,001
Gardnerella	-3,460b	0,001
Gemella	-3,246b	0,001
Pelomonas	-3,296c	0,001
Peptostreptococcus	-3,180c	0,001

Sphingobium	-3,416b	0,001
Acinetobacter	-3,798b	0
Actinomyces	-5,819b	0
Bacteroides	-5,444b	0
Bifidobacterium	-4,735b	0
Burkholderia	-5,877b	0
Clostridium	-5,343b	0
Companilactobacillus	-4,457c	0
Deinococcus	-4,783b	0
Erysipelothrix	-5,069b	0
Facklamia	-4,457c	0
Fenollaria	-4,703c	0
Fermentimonas	-3,621b	0
Fusobacterium	-3,644b	0
Glaciecola	-4,782b	0
Gottschalkia	-4,946b	0
Lactobacillus	-3,769b	0
Limosilactobacillus	-3,621c	0
Macrococcus	-5,777c	0
Megasphaera	-4,920b	0
Murdochiella	-5,796b	0
Neisseria	-4,257b	0
Oligotropha	-3,920b	0
Paenibacillus	-4,902b	0
Parvimonas	-4,408b	0
Propionimicrobium	-3,824b	0
Rhodopseudomonas	-3,548b	0
Salmonella	-3,509b	0
Schaalia	-4,323b	0
Sinimarinibacterium	-3,920b	0
Spirochaeta	-5,511b	0
Sporanaerobacter	-4,825b	0
Steroidobacter	-3,512b	0
Suicoccus	-4,637b	0
Tepidanaerobacter	-5,841b	0
Thermoanaerobacterium	-5,086b	0
Thiomicrospira	-4,286b	0
Trueperella	-5,261b	0
Varibaculum	-5,159c	0
Winkia	-4,457c	0
Xylanimonas	-4,286b	0
